# Acute Angle Closure Glaucoma Secondary to Suprachoroidal Hemorrhage Triggered by Valsalva Maneuver: A Rare Case

**DOI:** 10.7759/cureus.90401

**Published:** 2025-08-18

**Authors:** Eleni Papafotiou, Konstantinos Flindris, Chrysa Chatzipetrou, Athanasios Kaliardas, Ioannis Koumpoulis, Ioannis Melissourgos

**Affiliations:** 1 Ophthalmology, General Hospital of Ioannina "G. Hatzikosta", Ioannina, GRC; 2 2nd Department of Ophthalmology, Medical School, Aristotle University of Thessaloniki, Thessaloniki, GRC

**Keywords:** acute angle closure glaucoma, b-scan ultrasonography, secondary glaucoma, suprachoroidal hemorrhage, valsalva maneuver

## Abstract

Spontaneous suprachoroidal hemorrhage is a rare ophthalmic emergency, typically associated with ocular surgery or trauma. However, it can occur spontaneously in anticoagulated elderly patients after a sudden rise in venous pressure, such as during a Valsalva maneuver. We report a rare case of acute angle closure glaucoma secondary to suprachoroidal hemorrhage triggered by severe vomiting in an anticoagulated patient.

An 84-year-old male with atrial fibrillation on apixaban was hospitalized for biliary colic. Following repeated vomiting episodes, he experienced sudden, painful vision loss in the left eye. Examination revealed corneal edema, a shallow anterior chamber, a mid-dilated non-reactive pupil, and markedly elevated intraocular pressure (IOP) of 50 mmHg. Imaging demonstrated dislocation of the intraocular lens and angle closure, while B-scan ultrasonography and infrared image confirmed bilateral dome-shaped choroidal elevations consistent with suprachoroidal hemorrhage. Maximal topical and systemic IOP-lowering therapy was initiated. Due to significant comorbidities and high thromboembolic risk, anticoagulation was continued, and surgical intervention was not pursued. The patient’s pain was gradually relieved, but visual function did not recover.

This case highlights a rare but vision-threatening complication of spontaneous suprachoroidal hemorrhage induced by a Valsalva maneuver in a high-risk patient. In this case, vomiting likely caused a sudden rise in intra-abdominal pressure, leading to rupture of fragile choroidal vessels. The resulting anterior displacement of intraocular structures caused secondary angle closure glaucoma. Prompt diagnosis with B-scan ultrasonography and aggressive medical management are critical, though visual prognosis remains poor in such cases. Surgical drainage is considered in select patients but was contraindicated here.

Clinicians should maintain a high index of suspicion for suprachoroidal hemorrhage as a potential cause of acute angle closure in elderly, anticoagulated patients presenting with sudden painful vision loss. Timely diagnosis with B-scan and careful individualized management are crucial, particularly when surgical options are limited by systemic risk factors.

## Introduction

Extensive spontaneous suprachoroidal hemorrhage in the absence of surgery or trauma is an extremely rare event and is described in isolated case reports. In patients with this occurrence, systemic anticoagulation therapy is often a contributing factor [1-7]. Other associations with spontaneous suprachoroidal hemorrhage have been described in the literature. These include hypertension, age-related macular degeneration, increasing age, significant vascular disease, retinal telangiectasia, and Valsalva maneuver [1-3,8,9,10].

The Valsalva maneuver occurs when a person forcefully exhales against a closed glottis, leading to increased intrathoracic and intra-abdominal pressure. This elevated pressure compresses the vena cava, reducing venous return to the right atrium and subsequently decreasing cardiac output. When the glottis is released, intrathoracic and intra-abdominal pressures return to baseline, resulting in a rapid increase in venous return and cardiac output. This rebound effect produces a sudden rise in both peripheral arterial and venous pressure [11]. In a Valsalva maneuver, a sudden increase in venous pressure may lead to vessel wall rupture by an apparently excessive pressure gradient across the vessel wall. Thus, various types of periocular hemorrhages have been reported, i.e., conjunctival, vitreous, retinal, and orbital [3].

A spontaneous suprachoroidal hemorrhage can result in angle closure glaucoma and corneal edema secondary to forward displacement of the iris lens diaphragm [6,12]. We present a case of acute angle closure due to spontaneous suprachoroidal hemorrhage secondary to a Valsalva maneuver.

## Case presentation

An 84-year-old male with a history of atrial fibrillation treated with apixaban, hypertension, and pseudophakia was hospitalized for biliary colic. During admission, he experienced multiple severe vomiting episodes. Hours later, he reported sudden painful vision loss in his right eye.

**Figure 1:**
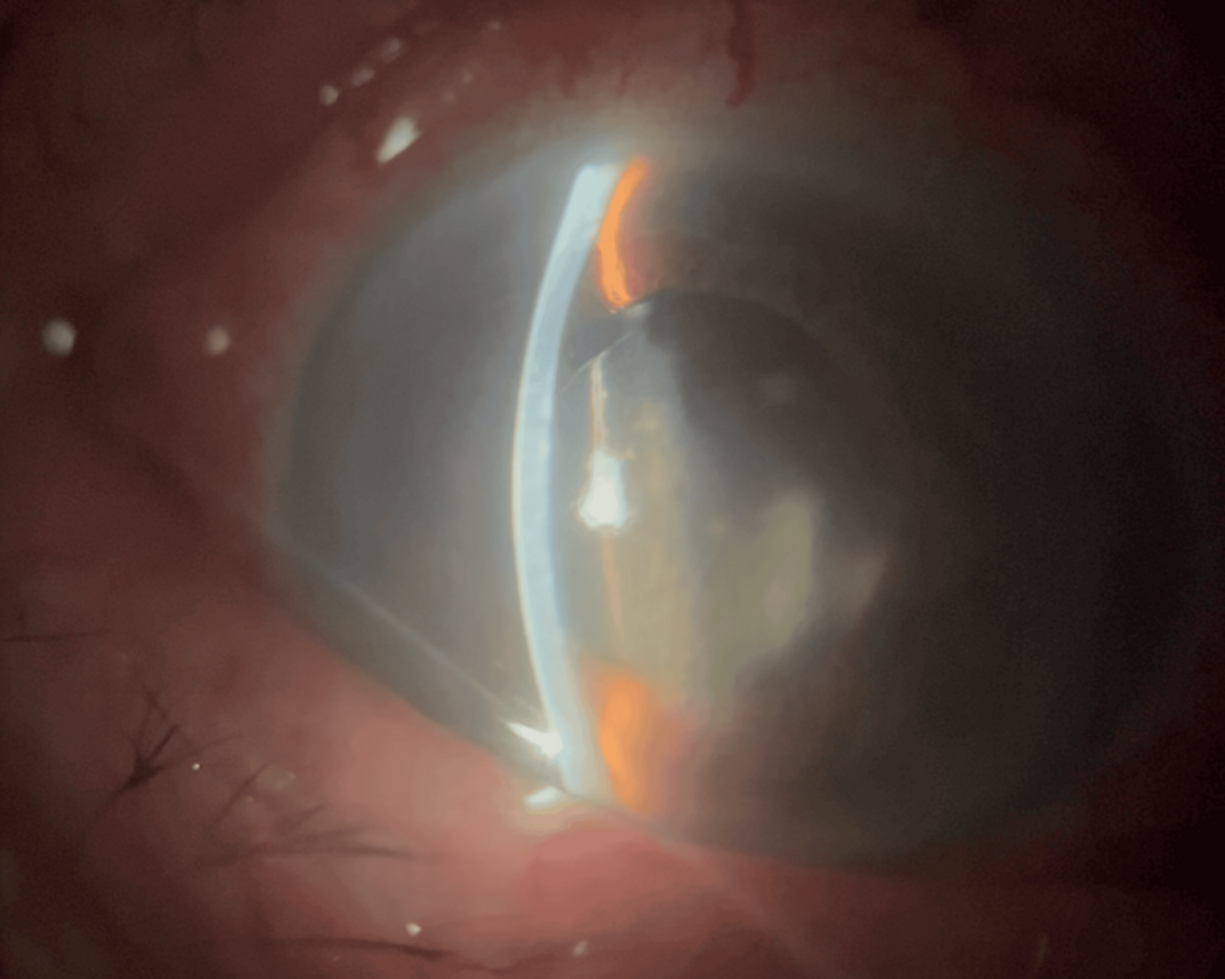
Slit-lamp photograph of the right eye demonstrating significant shallowing of the anterior chamber, corneal edema, a mid-dilated and fixed pupil, and inferior dislocation of the pseudophakic intraocular lens.

**Figure 2:**
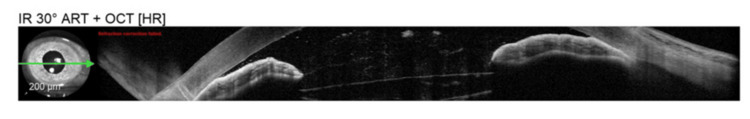
AS-OCT image showing appositional angle closure with the peripheral iris in direct contact with the corneal endothelium. AS-OCT, anterior segment optical coherence tomography

On examination, best corrected visual acuity in the right eye was hand movements. Intraocular pressure (IOP) was 50 mmHg. Slit-lamp examination revealed conjunctival injection, mild corneal edema, a shallow anterior chamber, and a mid-dilated non-reactive pupil (Figure 1). Notably, anterior segment findings included inferior dislocation of the pseudophakic intraocular lens, which contributed to anterior chamber shallowing. Gonioscopy, although limited due to mild corneal edema, and anterior segment optical coherence tomography (AS-OCT) demonstrated angle closure (Figure 2). The fellow eye was pseudophakic, with normal IOP and a deep, well-formed anterior chamber.

**Figure 3:**
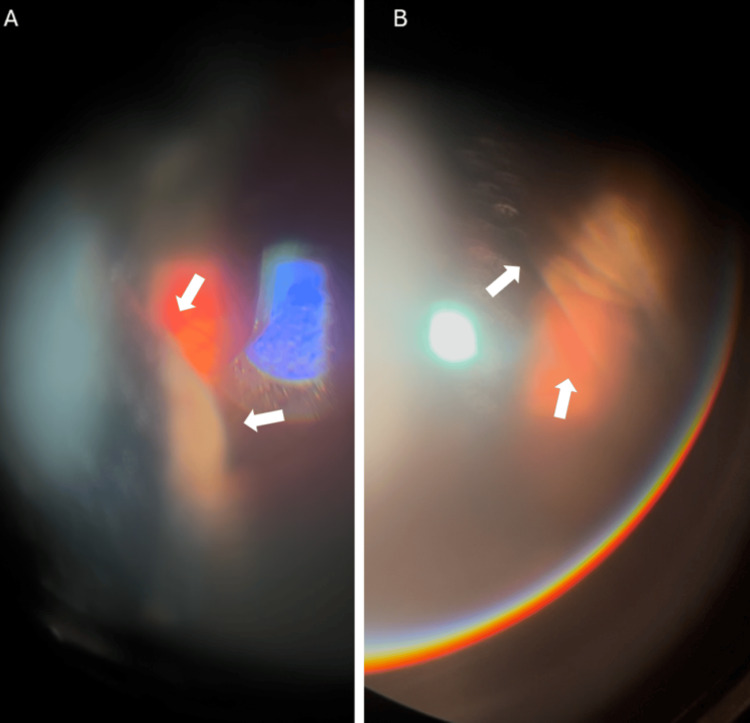
Fundus examination of the right eye showing (A) a dome-shaped dark choroidal elevation in the inferotemporal quadrant (white arrows) and (B) a dome-shaped choroidal elevation occupying the superonasal quadrant (white arrows).

**Figure 4:**
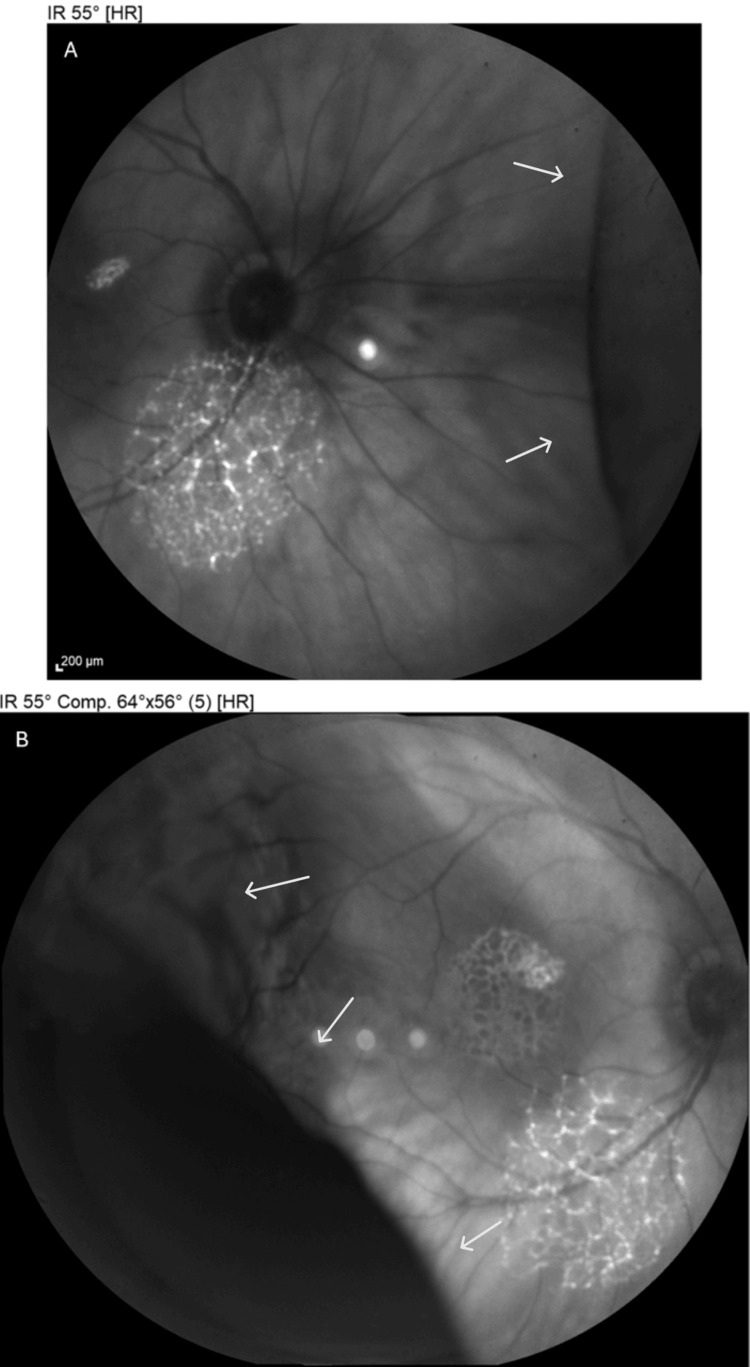
Infrared reflectance imaging of the right eye using SPECTRALIS® platform showing multiple dome-shaped dark choroidal elevations (white arrows) in the superonasal (A) and inferotemporal (B) quadrants.

Fundoscopic view was limited due to mild corneal edema, but partial visualization revealed two elevated, dark brown choroidal masses, located in the temporal half of the fundus and the other one in the nasal half on opposing sides, consistent with suprachoroidal hemorrhages (Figures 3A, 3B). Infrared reflectance imaging was also performed, revealing two well-defined, dome-shaped dark choroidal elevations in the inferotemporal and superonasal quadrants, consistent with localized suprachoroidal hemorrhages (Figure 4).

Maximal medical therapy was initiated with topical IOP-lowering agents (dorzolamide, brimonidine, and prostaglandin analog), topical dexamethasone eye drops, and IV mannitol at the maximal dosage. Acetazolamide was avoided due to borderline renal function. Following cardiology evaluation and a high CHA₂DS₂-VASc score, anticoagulation could not be withheld due to high cardioembolic risk from atrial fibrillation.

**Figure 5:**
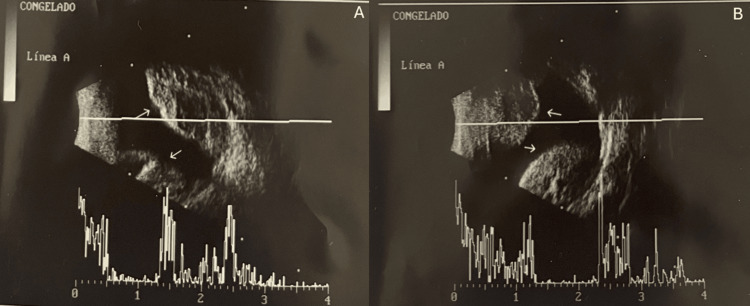
B-scan ultrasonography of the left eye showing a dome-shaped, hyperechogenic elevation (white arrows) in the posterior segment, consistent with suprachoroidal hemorrhage. The presence of opposing choroidal elevations (white arrows) suggests the characteristic “kissing choroidals.”

B-scan ultrasonography revealed a dome-shaped suprachoroidal mass consistent with hemorrhage (Figures 5A, 5B). The diagnosis of secondary acute angle closure glaucoma due to spontaneous suprachoroidal hemorrhage was made. The Valsalva maneuver from vomiting was considered the precipitating event.

Two days after presentation, IOP had decreased to 25-30 mmHg and remained at this level for the following two weeks, during which the patient reported significant pain relief. However, the suprachoroidal hemorrhage showed no signs of resolution and persisted for three weeks. The patient was referred to a specialized center, but given his multiple comorbidities and high surgical risk, no interventional procedure was performed. Visual function remained at hand motions, but pain was managed conservatively and ultimately resolved.

## Discussion

Spontaneous suprachoroidal hemorrhage is a rare but serious ophthalmic emergency typically associated with abrupt ocular hypotony following surgery or trauma. However, a subset of cases occurs without such events and is instead linked to systemic risk factors including anticoagulant use, advanced age, hypertension, and Valsalva maneuvers [7,10].

Our patient presented with classic risk factors: 84 years old, with atrial fibrillation on anticoagulant therapy, hypertensive, and recently subjected to repeated Valsalva maneuvers from vomiting due to biliary colic. The mechanical increase in intra-abdominal and intrathoracic pressure likely precipitated a rupture of fragile choroidal vessels. Similar mechanisms have been implicated in previous case reports describing suprachoroidal hemorrhage following bowel straining, coughing, or cardiopulmonary resuscitation [7,10].

Notably, the clinical presentation in our case included a shallow anterior chamber, mid-dilated fixed pupil, and “kissing choroidals” visualized on B-scan ultrasonography. The accumulation of blood between the choroid and sclera pushed the uveal tract forward, displacing the iris-lens diaphragm anteriorly and dislocating the pseudophakic intraocular lens, resulting in exacerbated anterior chamber crowding and angle closure. This is consistent with reports of secondary acute angle closure glaucoma resulting from forward displacement of intraocular structures by suprachoroidal blood accumulation [2,6,12-16].

The management of suprachoroidal hemorrhage, particularly spontaneous cases, remains complex and controversial due to its rarity and variable clinical course [1]. Initial therapy is almost universally conservative and includes IOP reduction through topical agents (e.g., beta-blockers, carbonic anhydrase inhibitors, alpha-agonists), systemic hyperosmotic agents such as intravenous mannitol, and cycloplegics to stabilize the anterior chamber. Corticosteroids, both systemic and topical, are commonly administered to control inflammation and reduce the risk of further hemorrhage [1,4,5].

Surgical intervention, typically in the form of delayed suprachoroidal drainage, is reserved for cases with persistent or appositional (“kissing”) choroidal detachments, intractable pain, or progressive vision loss. The timing of drainage is critical: most experts recommend waiting 10 to 14 days post-onset to allow clot liquefaction, minimizing the risk of further hemorrhage or iatrogenic retinal injury [4,5]. However, acute cases with uncontrolled IOP or severe pain may require earlier intervention. Some reports also describe adjunctive surgical approaches such as pars plana vitrectomy or cyclophotocoagulation, especially in cases refractory to drainage alone [3,5,15].

In our case, surgical options were contraindicated due to the patient’s advanced age, comorbidities, and ongoing anticoagulation, which could not be reversed due to a high risk of thromboembolic events from atrial fibrillation. Despite aggressive medical therapy, including maximal topical and systemic agents, the hemorrhage persisted, and vision could not be restored - an outcome consistent with the literature [7,10,13].

Overall, the visual prognosis of suprachoroidal hemorrhage, especially spontaneous or bilateral cases, remains poor. Even with timely drainage, many eyes progress to no light perception, and globe atrophy or phthisis bulbi may ensue. Therefore, early diagnosis, careful systemic management, and individualized surgical planning are essential to optimize outcomes [1,4,5,13].

## Conclusions

This case underscores the diagnostic and therapeutic challenges of secondary acute angle closure glaucoma due to spontaneous suprachoroidal hemorrhage in elderly, anticoagulated patients. While rare, this condition can be triggered by abrupt intra-abdominal pressure changes, such as those induced by the Valsalva maneuver. Clinicians must maintain a high index of suspicion in patients presenting with sudden painful vision loss, especially when systemic risk factors such as anticoagulation and hypertension are present.

Prompt diagnosis using B-scan ultrasonography is essential, as is early medical management to reduce IOP and control inflammation. However, visual prognosis remains guarded. Surgical intervention may be beneficial in selected patients but requires careful timing and risk stratification. Ultimately, this case highlights the importance of personalized treatment strategies and multidisciplinary collaboration when ocular emergencies intersect with complex systemic conditions.
